# Interaction-based discovery of functionally important genes in cancers

**DOI:** 10.1093/nar/gkt1305

**Published:** 2013-12-19

**Authors:** Dario Ghersi, Mona Singh

**Affiliations:** ^1^Lewis-Sigler Institute for Integrative Genomics, Princeton University, Princeton, NJ 08544, USA and ^2^Department of Computer Science, Princeton University, Princeton, NJ 08544, USA

## Abstract

A major challenge in cancer genomics is uncovering genes with an active role in tumorigenesis from a potentially large pool of mutated genes across patient samples. Here we focus on the interactions that proteins make with nucleic acids, small molecules, ions and peptides, and show that residues within proteins that are involved in these interactions are more frequently affected by mutations observed in large-scale cancer genomic data than are other residues. We leverage this observation to predict genes that play a functionally important role in cancers by introducing a computational pipeline (http://canbind.princeton.edu) for mapping large-scale cancer exome data across patients onto protein structures, and automatically extracting proteins with an enriched number of mutations affecting their nucleic acid, small molecule, ion or peptide binding sites. Using this computational approach, we show that many previously known genes implicated in cancers are enriched in mutations within the binding sites of their encoded proteins. By focusing on functionally relevant portions of proteins—specifically those known to be involved in molecular interactions—our approach is particularly well suited to detect infrequent mutations that may nonetheless be important in cancer, and should aid in expanding our functional understanding of the genomic landscape of cancer.

## INTRODUCTION

Understanding how the genetic and epigenetic alterations acquired during tumorigenesis give rise to specific cancer phenotypes represents a major aim of cancer biology, and is an important motivation for profiling human cancers at the genomic level. The Cancer Genome Atlas (TCGA) and the Cancer Genome Project have already generated vast amounts of information (1), and have opened up unprecedented opportunities for studying the functional consequences of the molecular alterations found in human cancers. Because of emerging technologies such as exome sequencing (2,3), characterizing human cancers at the level of proteins in large cohorts of patients has now become feasible, with the prospect of even more data at lower cost in sight (4). Analyzing these data sets represents a promising avenue for furthering our understanding of cancer and for ultimately obtaining better patient stratification, refined prognostic tools and novel therapeutic targets (5).

Although numerous mutations are usually observed in each cancer genome (6), it has been proposed that the majority of them play no role in tumorigenesis (5,7), even when focusing on the protein coding regions of biologically plausible candidate genes (8). The mutational landscape of cancer is described as dotted for the most part by ‘hills’ (infrequently altered genes, some of which are functionally important), with only a few ‘mountains’ (i.e. genes altered in a high percentage of cases) (6).

Therefore, a major challenge in cancer genomics is in distinguishing ‘driver’ genes—with an active role in tumorigenesis—from genes with ‘passenger’ mutations; this is especially difficult in the case of infrequently mutated genes that are nonetheless important in cancers (9). Further, systematic analyses of cancer genomes are necessary due to the high degree of molecular heterogeneity displayed by tumors, even among patients diagnosed with the same cancer type. In fact, cancer heterogeneity goes beyond inter-patient variability, as at least some tumors have been shown to contain distinct clones, with complex and shifting dominance hierarchies (10,11). It has also been argued that mutations that are neutral with respect to the initial tumorigenesis may affect the way a patient will respond to a treatment, or the evolution of the disease in later stages (9).

Here we introduce an approach for uncovering genes that play a functional role in cancer by focusing on the distribution across patient samples of missense mutations in the nucleic acid, small molecule, ion and peptide binding sites of the proteins they encode. Because proteins accomplish most of their functions by interacting with other molecules, the residues that participate in these interactions and comprise their binding sites represent critical functional regions. The motivation behind our method is that if a binding site has an enriched number of mutations across patient samples as compared with the total number of mutations uncovered for the entire protein, it may play a functionally important role in cancer even if the protein itself is not frequently mutated overall.

A small number of previous studies have confirmed the critical functional impact of cancer somatic mutations on binding sites (12–15). Further, a number of tools have been developed to distinguish passenger from driver mutations by screening for genes with high mutation rates (16–18), by training classifiers for this task (19–22) or by applying methods developed for assessing the functional impact of SNPs (23–29). Other approaches exploit patterns of conservation in sequence alignments (30), or take into account the specific tolerance to variation exhibited by functional groups of genes (31). The distinct topological properties of cancer genes in protein interaction networks provide another discriminating feature for ‘true’ cancer genes (32–34), as does the gain or loss of phosphorylation sites (35,36), or aggregated mutation data at the level of individual domains (37). Homology modeling of protein–protein interactions has also been proposed as a way to discover novel cancer-related genes (38). Further, some methods integrate multiple features in a probabilistic framework (39), whereas others exploit pathway information (40–42). Despite a wealth of previous studies, a large-scale comprehensive analysis of the distribution of cancer mutations with respect to protein binding sites is still lacking.

We aim to fill this gap by providing a fully automated pipeline for mapping missense mutations across cancer (and other types of) exomes, and analyzing their distributions with respect to protein–nucleic acid, protein–peptide, protein–small molecule and protein–ion interactions. We show that this pipeline can map a significant fraction of human proteins onto structures with annotated binding information. We next demonstrate that binding site residues are more frequently affected by cancer missense mutations than are other residues, but are less affected by single nucleotide polymorphisms (SNPs) observed across populations. Finally, we show that by focusing on binding sites, our approach identifies many genes already known to be causally implicated in cancers. Our software is available online at http://canbind.princeton.edu as a web server to explore the data deposited in TCGA, and as a standalone package to study newly sequenced genomes.

## MATERIALS AND METHODS

### Reference sequence data sets

Somatic missense mutations were obtained from TCGA. Eight cancer types with unrestricted data (as of March 2013) were used for this analysis: breast cancer (BRCA, 775 samples), clear cell kidney cancer (KIRC, 219 samples), colon adenocarcinoma (COAD, 84 samples), endometrial cancer (UCEC, 247 samples), glioblastoma multiforme (GBM, 290 samples), lung squamous carcinoma (LUSC, 176 samples), ovarian cancer (OV, 151 samples) and rectal adenocarcinoma (READ, 38 samples).

Chromosomal coordinates of the missense mutations provided by TCGA were converted to protein sequence coordinates by building the transcripts according to the Genome Reference Consortium assembly (GRCh37.p10) and by mapping the mutated codons to the corresponding position in protein sequence space. For each gene, mutation data were mapped to all the isoforms reported in the assembly, and the isoform that allowed the largest number of mutated positions to be mapped was selected. In the case of ties, the first isoform reported in the Genome Reference Consortium assembly was retained. We refer to these protein sequences as ‘reference’ sequences. There are 17 556 reference protein sequences with a missense or synonymous mutation in at least one of the eight cancer data sets. Additionally, SNP data, already mapped to reference transcripts, were obtained from the Single Nucleotide Polymorphism Database (dbSNP) repository (43), release 137.

### Assigning binding information to reference sequences

Binding information for structures deposited in the Protein Data Bank (44) was obtained from BioLip (45), a repository of biologically relevant protein–ligand interactions. We considered all the different types of interactions reported in BioLip (DNA/RNA, peptides, metals and small molecules), and extracted the sequences of the protein chains for which binding information was available. To increase coverage, we used the redundant version of BioLip (45).

The protein chain sequences were converted into a BLAST database using the BLAST +2.2.26 suite (46). For each reference sequence under consideration, we ran a BLASTP search with default parameters and an E-value <10^−6^. All hits with sequence identity >60% and coverage of the matching structure >80% were aligned to the initial reference sequence using Clustal Omega with default parameters (47). Using these pairwise alignments, the binding information contained in BioLip was transferred to the reference sequence only if the sequence identity in the binding residues was >90%.

We note that with the procedure outlined above, the same structure can map to multiple reference sequences, and more than one structure can map to the same reference sequence. To deal with this issue, for each reference sequence with multiple matching structures, we merged the mapped binding sites if they had at least one residue in common. Multiple binding sites within the same sequence with no residue in common were treated independently.

### Analyzing the distribution of cancer mutations and SNPs in protein binding sites

For both TCGA and dbSNP sets, we calculated (i) the total number of positions with structural information, *N*; (ii) the total number of binding residues, *K*; (iii) the total number of positions with a mutation (or SNP, respectively) in a structural part, *n*; and (iv) the total number of binding positions hit by a mutation (or SNP, respectively), *k*. We then calculated the probability of observing a given number of positions affected by a cancer mutation (SNP, respectively), using the hypergeometric distribution, and assuming that affected binding positions have the same probability of being mutated or affected by a SNP as non-binding positions. The probability density curves shown in Figure 2 were obtained from the hypergeometric distribution:
(1)
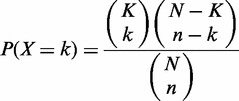

by using *K*, *N* and *n* from each data set, and varying the parameter *k*. The same procedure was followed to generate probability distributions for each type of binding site (Figure 2). *P*-values for the observed *k* were computed as 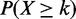 for cancer missense mutations and 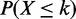 for SNPs.

### Assigning binding scores to protein sites

To capture the relative importance of a given residue within a binding site, we devised a score that uses the fraction of all heavy atoms of the residue within 4 Å of any ligand atom as a weight. In case of multiple structures matching the same TCGA sequence, the per-residue score was averaged over all the structures. That is, the binding score *b* for residue *i* in a given TCGA sequence was computed as:
(2)
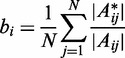

where *N* is the number of structures that match the sequence at position *i*; *A_ij_* is the set of heavy atoms of residue *i* in structure *j*; and 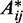 is the subset of atoms of *A_ij_* within 4.0 Å of any ligand atom. All binding residues received a score between 0 and 1.

### Selecting significantly mutated binding sites

For each human reference sequence with at least one reported mutation in a binding residue, we calculated the score *s_i_* of each residue by multiplying its binding score *b_i_* [see Equation (2)] by the number of samples with a reported missense mutation in that residue. We then summed these *s_i_* scores over all the mutated residues in the binding site, obtaining the total score per binding site *s_b_*. Next, we calculated the total number of mutations *m* within the sequence that affected residues with structural information, and to calculate empirical *P*-values, we generated 100 000 replicates, where for each replicate *m* residues with structural information are uniformly sampled with replacement. Finally, for each replicate, we calculated the binding score (as described above) using the sampled mutational data, and computed empirical *P*-values by counting the fraction of random samples with a more extreme score than that observed in the real case. In sequences with more than one binding site, we considered each site independently. We note that in the case of identical scores for all binding residues, this procedure would approximate sampling from a binomial distribution.

In Figure 3, we aggregated the mutations for all cancer types, whereas in Figure 4 we considered each cancer type separately. *P*-values of all binding sites in each cancer type were converted into false discovery rates (FDRs) using the Benjamini–Hochberg procedure (48).

## RESULTS

**Figure 1.**
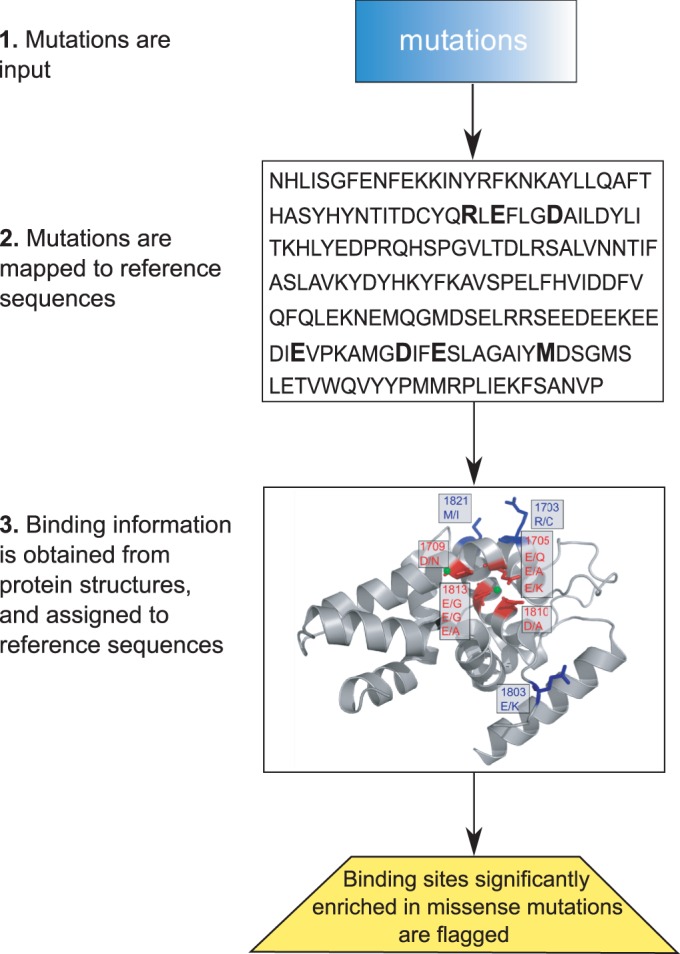
Schematic representation of the pipeline. Our computational pipeline to integrate sequence and structural information to identify genes whose encoded proteins have an enriched number of mutations in their binding sites proceeds as follows. First, mutations are mapped to a reference protein sequence. Second, information on protein binding is obtained from the Protein Data Bank (44) using the BioLip (45) database, and mapped onto the reference sequences. Then, mutations within each protein are statistically analyzed for their propensity to hit residues involved in binding with DNA, RNA, peptides, small molecules or ions.

We developed a computational approach (shown in Figure 1) that (i) takes as input mutations uncovered in exomes; (ii) maps them onto reference protein sequences; (iii) determines for each reference sequence if it is possible to transfer structural information from BioLip (45), a semi-manually curated database of protein–ligand interactions; (iv) extracts residues in the reference sequences that comprise binding sites for small molecules, DNA, RNA, peptides or ions, if structural information is available; (v) highlights genes with mutations that fall into a binding site of the corresponding proteins; and (vi) uncovers genes whose proteins are significantly enriched in mutations in at least one of their binding sites.

We applied this procedure to eight fully-available cancer data sets in TCGA, as well as to SNP data available from dbSNP, a catalog of both common and rare variants in nucleotide sequences. The cancer data sets consist of 1980 patient samples from either BRCA, KIRC, COAD, UCEC, GBM, LUSC, OV or READ.

### More than 20% of human genes can be mapped to protein structures that have binding information

Given the increasing number of cancer genomes that are being sequenced, it is likely that a cancer mutation will eventually be observed in almost every human gene; thus, we first set out to determine how many human genes can be mapped to protein structures with binding information. We gathered all the Uniprot protein sequences for *Homo sapiens* (May 2013); this yielded 88 817 protein sequences, which mapped to 20 421 genes. For each Uniprot sequence, we performed a BLAST search against the BioLip database, and found that 4471 human genes (21.9%) are similar to at least one BioLip entry (E-value <10^−6^, coverage of the structural part >80% and sequence identity >60%).

In the TCGA data set, we found 17 379 genes with at least one missense mutation in at least one of the eight cancer types that we included in the analysis (see ‘Materials and Methods’ section for more details). Of these 17 379 genes, 3943 (22.7%) were similar to a BioLip entry, using the same criteria as above. When requiring ≥90% sequence identity in the binding sites between the human sequence and the protein structure, the number of mappable genes went down to 3656 (21%).

### Common polymorphisms and cancer missense mutations show opposite trends in their propensity to affect protein binding sites

As a proof of concept, we first addressed the question of whether cancer missense mutations show a different propensity to affect protein binding sites as compared with SNPs obtained from dbSNP (43). In addition to missense mutations, we also considered synonymous mutations observed in TCGA.

**Figure 2.**
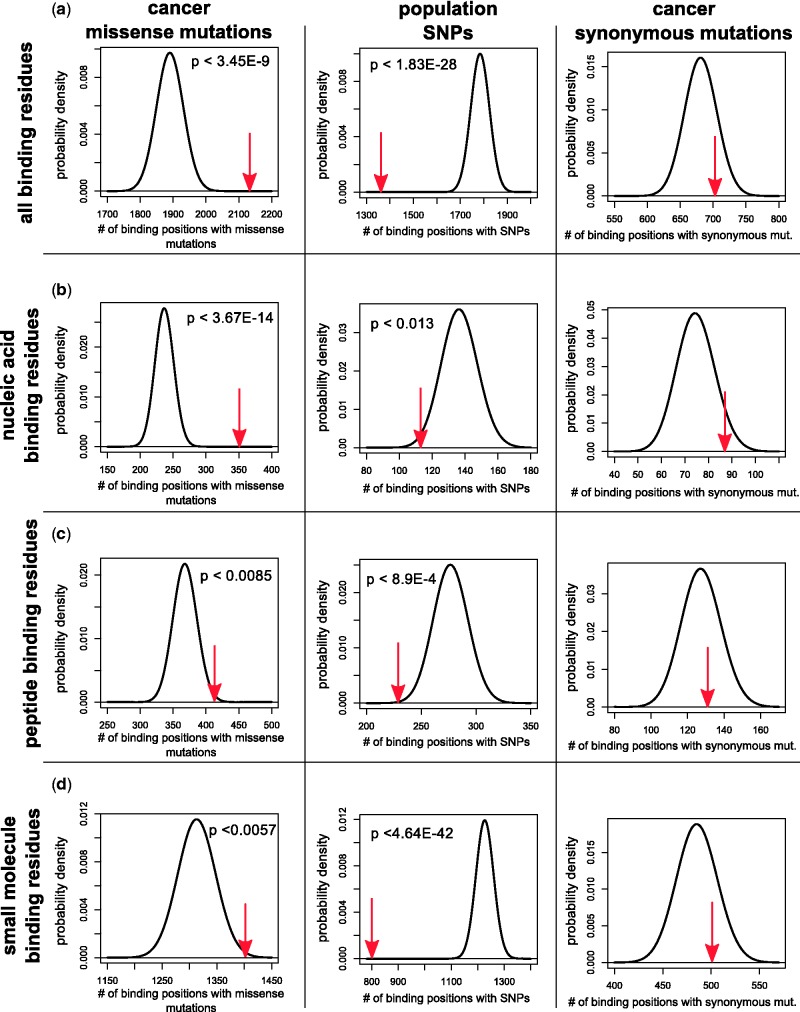
Cancer mutations and SNPs show opposite trends in their propensity to hit protein binding sites. The number of observed SNPs, cancer synonymous and cancer missense mutations (red arrows) as compared with the theoretical distributions for (**a**) all binding residues; (**b**) nucleic acid binding residues; (**c**) peptide binding residues; and (**d**) small molecule and ion binding residues. Theoretical distributions were computed using the hypergeometric distribution, under the null-hypothesis that binding residues have the same probability of being affected by SNPs or mutations as non-binding residues (see Materials and Methods). The *P*-values inside the panels (for SNPs and cancer missense mutations) of obtaining a value at least as extreme as the one observed were computed using the hypergeometric test.

We computed the total number of binding residues where cancer missense mutations, cancer synonymous mutations and population SNPs were observed. For this part of the analysis, only the number of affected positions (and not the frequency with which they were affected) was considered. An excess of cancer missense mutations was found in protein binding sites (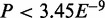, hypergeometric test, Figure 2a, left). In contrast, SNPs tended to avoid protein binding sites (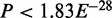, Figure 2a, middle). We hypothesized that if cancer missense mutations are enriched in binding sites because they play a functional role, we should not find this enrichment when considering the distribution of synonymous mutations found in the same cancer types. As expected, the total number of synonymous mutations in protein binding sites lay within the expected range (Figure 2a, right).

To determine whether the same trend could be observed for each type of binding sites, we repeated the analysis by considering in turn each of (i) nucleic acid binding residues, (ii) peptide binding residues and (iii) small molecule and ion binding residues. The global trends observed with aggregate binding data were recapitulated for each of the three types of binding sites, with cancer missense mutations hitting more than expected by chance nucleic acid binding residues (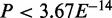, Figure 2b), peptide binding residues (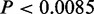, Figure 2c) and small molecule and ion binding residues (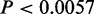, Figure 2d). In contrast, SNPs were less likely than expected to hit nucleic acid binding residues (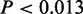, Figure 2b), peptide binding residues (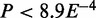, Figure 2c) and small molecule and ion binding residues (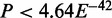, Figure 2d).

We also set out to determine whether the same conclusions held when considering the frequency with which binding positions were affected. Because dbSNP does not directly provide the number of times a SNP was observed, we limited this analysis to cancer missense and synonymous mutations. We computed the mutation frequency in binding and non-binding residues and performed a binomial test, defining the probability of randomly hitting a binding residue as the fraction of residues with structual information that are binding residues. Across our entire data set, the probability of observing at least the observed number of cancer missense mutations in binding residues by chance alone was 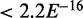, whereas the probability of observing at least the observed number of cancer synonymous mutations in binding sites by chance alone was 0.28.

It is important to point out that the type of analysis shown in Figure 2 only reflects global tendencies, and biologically important exceptions for specific genes can be observed. For example, while SNPs tended to fall outside protein binding sites, we found that genes belonging to the major histocompatibility complex had between two and three times more binding positions with SNPs than expected, confirming the well-known allelic diversity of major histocompatibility complex genes (49). No other genes had more than two times the binding positions with SNPs than expected.

### Genes that are significantly mutated in protein-binding sites are enriched in well-known cancer genes

Having confirmed that cancer missense mutations tend to occur in protein binding sites more frequently than expected, we set out to identify genes with a statistically significant excess of mutations in binding sites across the TCGA data, aggregating the mutations found in the eight cancer types. For each binding site, we calculated a mutation score, defined as the sum across the positions that comprise it of the number of mutations affecting each position weighted by the binding score of the position. The binding score is calculated as the fraction of all the heavy atoms of the residue in that position in the mapped structure that are in proximity (≤4.0 Å) of the ligand. We then computed an empirical *P*-value for each binding site using a permutation test (see ‘Materials and Methods’). We note that by using data obtained exclusively from large-scale DNA sequencing, we minimize the risk of study biases that would artificially inflate the mutation count for well-characterized binding sites.

**Figure 3.**
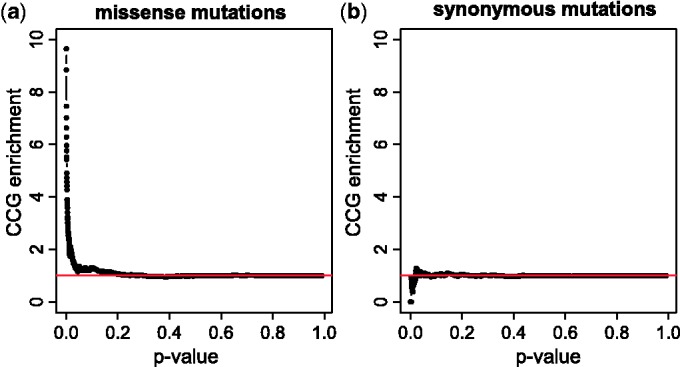
Genes whose encoded proteins are significantly mutated in binding sites are enriched in well-known cancer genes. The enrichment of CCGs at given *P*-value thresholds, computed using (**a**) missense mutations and (**b**) synonymous mutations. For each *P*-value threshold, we obtained the set of genes whose proteins have at least one binding site with a mutation enriched at this level of significance and computed the enrichment as the ratio between the fraction of CCGs in the genes at the given *P*-value threshold and the fraction of CCGs in the whole set of genes. The results shown here were obtained by aggregating the mutations observed across all eight cancer types.

To validate the method in the absence of a gold standard, we computed the enrichment for Cancer Census Genes (CCGs)—a curated list of genes causally implicated in cancer (50)—in gene sets with progressively smaller *P*-values. The results showed that smaller *P*-value thresholds yielded increasingly higher CCGs enrichment values (Figure 3). For example, 39 genes were in the top 2.5% of the *P*-value distribution, and 10 of them were CCGs; because the total number of binding sites with at least one mutation in a binding site was 1379, of which 104 were in CCGs, this is an enrichment factor of >3.40 (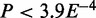, hypergeometric test). As a control, we repeated the analysis on synonymous cancer mutations, obtaining—as expected—no enrichment in CCGs for small *P*-values (Figure 3).

### Per-cancer type analysis

The analysis described above was carried out by considering all the reported somatic mutations in the eight cancer types together. Although the results suggest that genes whose corresponding proteins are significantly affected by mutations in their binding sites are enriched in those with a known role in tumorigenesis, they do not reveal what happens at the level of individual cancer types.

**Figure 4.**
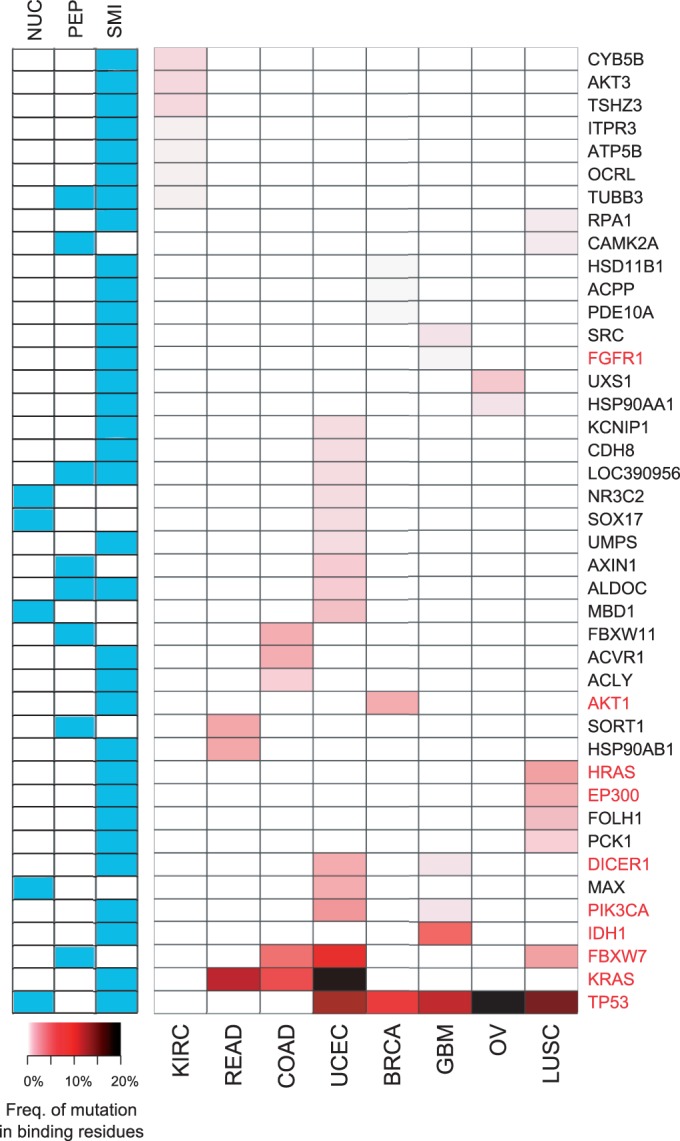
Genes whose proteins are significantly mutated in binding residues (per cancer type). Genes in each of the eight cancer types with FDRs < 0.1 are shown and colored according to their frequency of mutation in the binding residues of their encoded proteins, normalized by the number of samples. The frequency of mutation in binding residues is simply the fraction of samples with a mutation in a binding residue. The left panel shows the different types of binding sites within the protein that are found to have mutations (NUC: nucleic acid binding site; PEP: peptide binding site; and SMI: small molecule and ion binding site). CCGs [50] are highlighted in red. BRCA: breast cancer; KIRC: clear cell kidney cancer; COAD and READ: colon and rectal adenocarcinoma; UCEC: endometrial cancer; GBM: glioblastoma multiforme, LUSC: lung squamous carcinoma; and OV: ovarian cancer.

To address this question, we carried out a per-cancer type analysis, using only the mutations reported in each cancer type. Using an FDR < 0.1, we obtained 42 genes whose encoded proteins were significantly mutated within binding sites in at least one cancer type. Figure 4 shows the significant genes, with the total number of mutations in binding residues normalized by the number of samples. As expected, most of the CCGs appeared to be mutated at a higher frequency in multiple cancer types, whereas other genes displayed more cancer-specific patterns.

The contribution of different types of binding sites is also shown in Figure 4. We note that while mutations observed in small molecule or ion binding sites far outnumber the mutations in other binding sites, these binding sites were also the most highly represented in our structural data set. A small molecule or ion binding site was found in >82% of the structural entries, whereas nucleic acid and peptide binding sites were found in 14 and 23% of the entries, respectively, with some proteins having more than one type of binding site.

We performed Gene Ontology (GO) (51) biological process enrichment analysis on this set of 42 genes. Using a Bonferroni corrected *P*-value threshold of <0.05, the most specific GO-enriched terms were fibroblast growth factor receptor signaling pathway, epidermal growth factor receptor signaling pathway, response to insulin stimulus, neurotrophin TRK receptor signaling pathway and phosphate-containing compound metabolic process; we note that these terms are mostly related to signaling.

The original publications for seven of the eight cancer types considered here reported genes with significantly recurrent mutations (Supplementary Text S1). As expected, there was agreement between our approach and the list of recurrently mutated genes for several well-known players (e.g. AKT1, FBXW7, KRAS, PIK3CA and TP53). However, by focusing on protein-binding sites, our approach was able to uncover genes that were not detected as recurrently mutated, a few of which are discussed further below.

### Examples of genes significantly mutated in binding sites

To showcase how our computational pipeline may assist in analyzing cancer proteomes, we briefly highlight a few proteins that were found to have an enriched number of mutations in their binding sites across cancer proteomes.

#### DICER1

**Figure 5.**
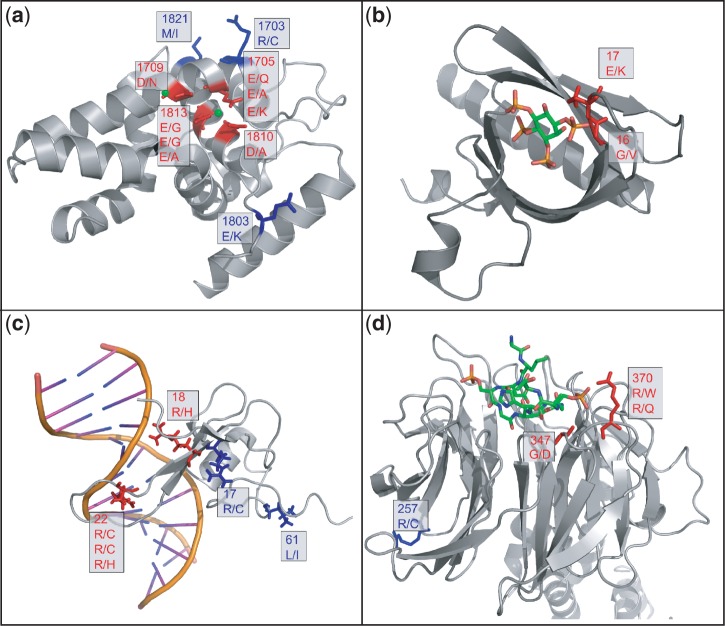
Examples of proteins significantly mutated in binding residues. (**a**) RNase IIIb domain of DICER1[PDB code: 2eb1 (52)]. Six of the nine mutations that fall in the RNase IIIb domain of DICER1 in ‘endometrial cancer’ and two of the two mutations in ‘glioblastoma multiforme’ affect a negatively charged valley involved in magnesium binding (52). Magnesium ions are required for the catalytic activity of DICER RNase IIIb domain (52,53). Binding and non-binding residues are colored in red and blue, respectively. (**b**) Pleckstrin homology domain of AKT3 with mutations in ‘clear cell kidney cancer’ [PDB code: 1h10 (54)]. Both of the observed mutations fall in proximity to the binding site for phosphatidylinositol (3,4,5)-trisphosphate. (**c**) MBD1 (in complex with DNA) with mutations in ‘endometrial cancer’ [PDB code: 1ig4 (55)]. Four of the six mutations replace two arginines in proximity to DNA. (**d**) FBWX11 (in complex with beta-catenin) with mutations in ‘colorectal cancer’ and ‘glioblastoma multiforme’ [PDB code: 1P22 (56)]. The arginine mutation at 370 was found in both colorectal cancer (to glutamine) and glioblastoma multiforme (to tryptophan). The mutation at 257 from arginine to cysteine outside the binding site was found in colorectal cancer.

Our approach suggests a role for DICER1 in endometrial cancer (Figures 4 and 5a), where six of the nine observed mutations cluster around two Mg2+ binding sites in the RNase IIIb domain. Mg2+ ions have been shown to play a role in DICER’s ability to bind RNA and cleave it (52), and are found in a negatively charged valley (Figure 5a). The six mutations in endometrial cancer replace the negatively charged amino acids (Asp and Glu) with either positively charged (Lys) or non-charged amino acids (Asn, Gly and Ala), thereby suggesting a potential loss of Mg2+ binding and RNase activity.

Supporting the potential role of DICER in cancer, we note that global microRNA (miRNA) downregulation is frequently observed in human cancers (57). Work by Martello *et al.* showed that the miRNA family miR103/107 (over-expressed in some breast cancers) can lead to less-differentiated cancer cells and a metastatic phenotype by targeting DICER, a crucial component of miRNA processing (58). More recently, recurrent somatic mutations of DICER1 around metal-binding residues were found in non-epithelial ovarian cancer (59).

#### AKT3

AKT consists of three isoforms (AKT1, AKT2 and AKT3) encoded by distinct genes but each containing a pleckstrin homology domain (60). AKT3—a gamma serine/threonine kinase in the phosphatidylinositol 3-kinase pathway—has only two mutations in clear cell kidney cancer (Figure 4). However, both these mutations (Gly16Val and Glu17Lys) fall in the binding site for phosphatidylinositol (3,4,5)-trisphosphate, in the pleckstrin homology domain of the kinase (Figure 5b).

It has previously been found that over-expression of AKT3 is a critical factor that correlates with cell proliferation in ovarian cancer (61). The Glu17Lys mutation seen in the binding site of AKT3 in kidney cancer was also found in the pleckstrin homology domain of AKT1 in breast, colorectal and ovarian cancers, and results in the activation of AKT1, followed by downstream signaling and cell transformation (62). The same gain-of-function mutation in AKT1 was also subsequently observed in squamous lung cancer, with a frequency of 0.6% (63).

#### MBD1

Methyl-CpG-binding domain protein 1 (MBD1) is a transcriptional repressor that functions by binding CpG islands in gene promoters (64). MBD1 has been shown to bind the promoters of known tumor suppressor genes (e.g. p16, VHL and E-cadherin) (64). Our pipeline found MBD1 to be significantly mutated in binding sites in the endometrial cancer data set (Figure 4) because of four somatic mutations affecting a binding site that recognizes methylated DNA (Figure 5c). The mutations replace two Arg residues in positions 18 and 22 with either His or Cys. Interestingly, another mutation (Arg17Cys) that falls just outside the binding site (>4 Å from DNA) but is next to Arg18 has been associated with Rett syndrome in the MeCP2 gene, which has an identical MBD domain (55).

#### FBXW11

FBXW11 (also known as HOS) is part of the SCF complex, which mediates the proteasome-dependent degradation of phosphorylated substrates (65). We found FBXW11 to be significantly enriched in binding site mutations in colon adenocarcinoma (FDR < 0.1), and of borderline significance in glioblastoma multiforme (FDR = 0.104) (Figure 4). Three of the four mutations observed in the two cancer types occur in proximity to a site that binds beta-catenin (Figure 5d), a well-studied protein involved in the Wnt signaling pathway, and implicated in several malignancies, such as colon cancer, melanoma, medulloblastoma and others (66). In the complex shown in Figure 5d, Arg370 is 2.4 Å away from a phosphorylated Ser in beta-catenin, and is replaced with a Gln in colon adenocarcinoma and a Trp in glioblastoma multiforme. Another mutation in colon adenocarcinoma affects Gly347, which is replaced with a negatively charged amino acid (Asp). We note that mutations in beta-catenin, FBXW11’s substrate, have also been observed in colon cancer, where they affect Ser and Thr residues that are essential for the phosphorylation-dependent degradation of beta-catenin (67).

## DISCUSSION

Owing to the growing catalog of molecular events that occur during tumorigenesis, cancer biology has reached a turning point. A high resolution view of the mutational landscape in cancer is now becoming available and—as a consequence—almost every gene has been or will be found to be mutated in at least a few patient samples.

In this article, we have described a novel structural bioinformatics approach that aims to understand the effects of mutations in the broader context of a protein’s molecular interactions, and to assess the potential of mutations to disrupt these interactions. Our pipeline represents a complementary approach to existing methods, as it directly uses structural information in the context of large-scale cancer resequencing data, and is a step toward providing mechanistic interpretations of the effects of mutations. One important aspect of our approach is that it can highlight genes that may be infrequently mutated overall, but for which mutations preferentially occur in binding sites. It has previously been observed that genes that are mutated at low frequency can play important functional roles in cancer, and account (at least in part) for the high degree of clinical heterogeneity observed in many cancer types (10,11).

Given the complexity and diversity of cancer proteomes, it is not surprising that many computational methods to prioritize candidate genes have been developed over the years. Perhaps most similar to the work described here are earlier attempts to combine structural information about protein binding sites with cancer mutation data. Stehr *et al.* (14) characterized the structural differences between oncogenes and tumor suppressors, and their analysis included protein functional sites. However, their aim was to describe the structural impact of mutations in well-known and frequently mutated genes, and thus they analyzed 24 well-characterized genes. In contrast, our method analyzes thousands of genes and identifies those that may be infrequently mutated overall, but are nonetheless important. A more recent paper by Nishi *et al.* (15) studied the changes in binding energy caused by mutations in glioblastoma multiforme and further included protein–protein interfaces. Our method does not attempt to estimate the impact of any individual mutation on the binding energy, but simply highlights genes whose mutation patterns statistically deviate from expectation, thereby suggesting a selective process at work. The power of our method lies in its ability to detect genes with only a few mutations concentrated in a small functional part of the protein (i.e. its binding sites). For this reason, we excluded protein–protein interfaces from our analysis, which can contain a substantial fraction of a protein’s residues. For example, in the data set used in (15), on average, 41% of the residues within a protein take part in protein–protein interfaces (see Supplementary Text S2).

One limitation of our approach is that it requires structural information to accurately pinpoint the binding residues. As a consequence, a little over one-fifth of human genes can be studied at this time, although we expect this number to grow over time as our knowledge of protein structures increases. Moreover, we note that an excess of mutations in binding sites does not reveal whether a loss or a gain of function is at play, although it has been proposed that gain of function is more likely to occur in binding sites (14). Further work will be required to predict the functional implications at the cellular level of a mutational event in a binding site.

To conclude, by focusing on protein binding sites, we developed an automated approach that is particularly well suited to capture relatively rare mutations that are likely to perturb protein function. Therefore, our approach and the accompanying software should prove useful to the community as a hypothesis-generating tool, and as a bridge between detailed structural analyses of select genes and broad statistical screenings of cancer genomes.

## SUPPLEMENTARY DATA

Supplementary Data are available at NAR Online.

## FUNDING

American-Italian Cancer Foundation Postdoctoral Fellowship (to D.G.). National Science Foundation [ABI-1062371 to M.S.] (in part); National Institutes of Health [GM076275 to M.S.] (in part); National Institute of Health Center of Excellence [P50 GM071508 to David Botstein], (in part); Forese Family Fund for Innovation. Funding for open access charge: [ABI-1062371 and GM076275].

*Conflict of interest statement*. None declared.

## Supplementary Material

Supplementary Data
