# Supplementary material for: Interaction-based discovery of functionally important genes in cancers
Source: Nucleic Acids Res. 2013 Dec 19;42(3):e18. doi: 10.1093/nar/gkt1305 (PMC3919581; doi:10.1093/nar/gkt1305)
Supplement: Supplementary Data [file supp_42_3_e18__index.html]

Interaction-based discovery of functionally important genes in cancers — Interaction-based discovery of functionally important genes in cancers — Supplementary Data 

# Interaction-based discovery of functionally important genes in cancers

## Supplementary Data

files

**Files in this Data Supplement:**

- Supplementary Data - pdf file
