# Supplementary material for: Interaction-based discovery of functionally important genes in cancers
Source: Nucleic Acids Res. 2013 Dec 19;42(3):e18. doi: 10.1093/nar/gkt1305 (PMC3919581; doi:10.1093/nar/gkt1305)
Supplement: Supplementary Data [file supp_gkt1305_nar-02989-met-n-2013-File001.pdf]

Supplementary Material

Dario Ghersi and Mona Singh

Supplementary Text S1

Recurrently mutated genes in published large-scale studies. The genes in red are significantly mutated (FDR < 0.1) in the binding site. Genes with at least one mutation in a structural residue are underlined.

BRCA (*Nature.* **490** (7418):61-70) – AFF2, AKT1, CBFB, CCND3, CDH1, CDKN1B, CTCF, DCAF4L2, GATA3, GPR32, GPS2, HIST1H2BC, LEC19A, MAP2K4, MAP3K1, MLL3, NCOR1, NF1, OR6A2, PGR, PIK3CA, PIK3R1, PTEN, PTPN22, PTPRD, RB1, RUNX1, RYR2, SEPT13, SF3B1, TBL1XR1, TBX3, TP53, USH2A, ZFP36L1

COAD, READ (*Nature.* **487** (7407):330-337**)** – ACVR1B, APC, ATP6V0D2, BRAF, CASP8, CDC27, CTNNB1, DMD, EDNRB, FAM123B, FBXW7, FZD3, GPC6, GRIK3, KIAA1804, KRAS, MAP7, MIER3, MYO1B, NRAS, PIK3CA, PTPN12, SLC9A9, SMAD2, SMAD4, SOX9, TCERG1, TCF7L2, TP53, TTN, WBSCR17

GBM (*Nature.* **455** (7216):1061-1068) – EGFR, ERBB2, NF1, PIK3CA, PIK3R1, PTEN, RB1, TP53

KIRC (clean cell kidney cancer) – in press

LUSC (*Nature.* **489** (7417):519:525**)** – CDKN2A, HLA-A, KEAP1, MLL2, NFE2L2, NOTCH1, PIK3CA, PTEN, RB1, TP53

OV (*Nature.* **474** (7353):609-615**)** – BRCA1, BRCA2, CDK12, CSMD3, FAT3, GABRA6, NF1, RB1, TP53

UCEC (*Nature.* **497** (7447):67-73**)** – ARID1A, ARID5B, CTCF, CTNNB1, FBXW7, KRAS, PIK3CA, PIK3R1, PPP2R1A, PTEN, RPL22, TP53

Supplementary Text S2

To calculate the average fraction of residues in protein structures that are involved in protein-protein interactions, we downloaded the IBIS dataset used by Nish et al., (*PLoS One 2013*) from http://www.ncbi.nlm.nih.gov/Structure/ibis/ibis.cgi and selected the protein chains with a “PPI” (“protein-protein interaction”) annotation. We obtained 184,450 distinct chain identifiers. For each chain, we calculated the number of residues involved in a protein-protein interaction based on the annotation provided by IBIS, and extracted the total number of residues in the chain from the “pdb_seqres” file available from the Protein Data Bank (ftp://ftp.pdbj.org/pub/pdb/derived_data/pdb_seqres.txt). We then calculated the average per-chain fraction of residues involved in PPIs, and obtained a value of 41%.
